# SALL4 promotes gastric cancer progression via hexokinase II mediated glycolysis

**DOI:** 10.1186/s12935-020-01275-y

**Published:** 2020-05-24

**Authors:** Meng Shao, Jiayin Zhang, Jiahui Zhang, Hui Shi, Yu Zhang, Runbi Ji, Fei Mao, Hui Qian, Wenrong Xu, Xu Zhang

**Affiliations:** 1 Jiangsu Key Laboratory of Medical Science and Laboratory Medicine, School of Medicine, Jiangsu University, 301 Xuefu Road, Zhenjiang, Jiangsu 212013 China; 2 Department of Clinical Laboratory Medicine, The Affiliated People’s Hospital of Jiangsu University, Zhenjiang, Jiangsu 212002 China

**Keywords:** Gastric cancer, SALL4, Hexokinase II, Glycolysis, Progression

## Abstract

**Background:**

The stem cell factor SALL4 is reactivated in human cancers. SALL4 plays diverse roles in tumor growth, metastasis, and drug resistance, but its role in tumor metabolism has not been well characterized.

**Methods:**

The glycolytic levels of gastric cancer cells were detected by glucose uptake, lactate production, lactate dehydrogenase activity, ATP level, and hexokinase activity. QRT-PCR and western blot were used to detect the changes in the expression of glycolytic genes and proteins. The downstream target genes of SALL4 were identified by microarray. The regulation of hexokinase II (HK-2) by SALL4 was analyzed by luciferase reporter assay and chromatin immunoprecipitation assay. Transwell migration assay, matrigel invasion assay, cell counting assay and colony formation assay were used to study the roles of HK-2 regulation by SALL4 in gastric cancer cells in vitro. The effects of SALL4 on glycolysis and gastric cancer progression in vivo were determined by subcutaneous xenograft and peritoneal metastasis tumor models in nude mice.

**Results:**

SALL4 knockdown inhibited glucose uptake, lactate production, lactate dehydrogenase activity, ATP level and hexokinase activity in gastric cancer cells, and decreased the expression of glycolytic genes and proteins. Microarray analysis showed that SALL4 knockdown affected glycolysis-related pathway. The regulation of HK-2 gene expression by SALL4 was confirmed by luciferase reporter assay and chromatin immunoprecipitation assay. HK-2 knockdown abrogated the promotion of glycolysis by SALL4 in gastric cancer cells, indicating that HK-2 acts as a downstream effector of SALL4. Moreover, HK-2 knockdown reversed the promoting role of SALL4 in gastric cancer cell proliferation, migration and invasion, suggesting that SALL4 drives gastric cancer progression by upregulating HK-2.

**Conclusions:**

SALL4 promotes gastric cancer progression through HK-2-mediated glycolysis, which reveals a new mechanism for the oncogenic roles of SALL4 in cancer.

## Background

Tumor cells are characterized by abnormal glycolysis, accompanied by increased glucose uptake and lactate production, which is called aerobic glycolysis or Warburg effect [1]. Although the ATP produced by tumor glycolysis is not much, it can supply energy quickly and satisfy the high-speed proliferation of tumor cells. The intermediate products generated by glycolysis could be used to synthesize nucleic acids, amino acids and fats required for the proliferation of tumor cells and the acidic microenvironment caused by lactic acid could enhance tumor metastasis and therapy resistance [2]. Therefore, there is a close relationship between glycolysis and various malignant phenotypes of tumor cells.

Transcription factors play a crucial role in the regulation of aerobic glycolysis, such as hypoxia inducible factor-1α (HIF-1α), c-Myc, p53, and sine oculis homeobox 1 (SIX1) [3–7]. SALL4 is a zinc finger protein transcription factor that is crucial in the self-renewal and pluripotency of embryonic stem cells [8, 9]. With the maturation of tissues and organs, the expression of SALL4 is gradually down-regulated. In adult tissues, SALL4 can only be detected in germ cells and hematopoietic stem cells [10]. However, in many cancers including blood cancers and solid tumors, SALL4 expression is restored [11–15]. The previous studies have shown that SALL4 is involved in tumor cell proliferation, migration, invasion, DNA damage repair, and drug resistance, suggesting a high potential of SALL4 as tumor biomarker and therapeutic target [16–18].

Gastric cancer is one of the most common cancers worldwide and ranks second in tumor-related deaths [19, 20]. We have previously shown that SALL4 is highly expressed in gastric cancer and its upregulation is associated with lymph node metastasis and poor prognosis [14]. SALL4 could enhance the proliferation, migration and invasion of gastric cancer cells by regulating CD44, DANCR, and TGF-β1 expression [21–23]. There are few studies on the role of SALL4 in tumor metabolism. Kim et al. demonstrate that SALL4 promotes the ubiquitination of heterochromatin protein 1α (HP1α), which increases the expression of glucose transporter 1 (GLUT1) and hence promotes glycolysis [18]. Whether SALL4 could affect glycolysis through other mechanism still needs further study.

In the present study, we showed that SALL4 overexpression promoted while SALL4 knockdown inhibited glycolysis in gastric cancer cells. SALL4 promoted the glycolysis of gastric cancer cells by activating the expression of HK-2, a key rate-limiting enzyme of glycolysis. HK-2 knockdown reversed the promotion of gastric cancer cell proliferation, migration, and invasion by SALL4. Our results suggest that SALL4 could promote gastric cancer progression through HK-2-mediated glycolysis, which represents a new mechanism for the oncogenic roles of SALL4 in cancer.

## Materials and methods

### Cell culture

Human normal gastric mucosa epithelial cell line GES-1 was obtained from Gefan Biological Technology (Shanghai, China). Human gastric cancer cell lines HGC-27, AGS, SGC-7901 were purchased from the Institutes for Biological Sciences at the Chinese Academy of Sciences (Shanghai, China). Human gastric cancer cell line MGC-803 was obtained from the Cell Resource Center, Institute of Basic Medicine, Chinese Academy of Medical Sciences (Beijing, China). GES-1, HGC-27 and SGC-7901 cells were cultured in RPMI 1640 medium (Life Technologies, Carlsbad, CA, USA) with 10% fetal bovine serum (FBS; Life Technologies) at 37 °C in humidified air with 5% CO_2_. The other cell lines were cultured in high-glucose DMEM (Life Technologies) supplemented with 10% FBS. Cells have been regularly tested for Mycoplasma and are free of contamination.

### Gene transfection

The cells were seeded in 6-well plates at 50% density and incubated overnight. SALL4 overexpression in HGC-27 and AGS cells was achieved by using SALL4 expression plasmid (RC213089) and pCMV6-Entry vector plasmid (PS100001) purchased from OriGene (OriGene, Rockville, MD, USA). The overexpressing plasmids were transfected into the cells by LipoFiter transfection reagent (Hanbio, Shanghai, China) in a serum-free medium. The cells were changed to a complete medium at 6 h after transfection and cultured for another 42 h. Chemically synthesized SALL4 siRNAs and the matching scramble control siRNAs were purchased from Genechem Company (Shanghai, China). The siRNAs were transiently transfected into SGC-7901 and MGC-803 cells at a final concentration of 20 nM by using LipoFiter transfection reagent (Hanbio, Shanghai, China) in a serum-free medium. The cells were changed to a complete medium at 6 h after transfection and cultured for another 42 h. The SALL4-targeting shRNA lentivirus was provided by Genechem (Shanghai, China). MGC-803 cells were transfected with lentivirus at an MOI (multiplicity of infection) of 100 for 24 h and then selected with puromycin (0.8 µg/mL) for 3 days. The sequences of siRNAs and shRNAs were shown in Additional file 1.

### Luciferase reporter assay

MGC-803 cells or AGS cells were transfected with the luciferase reporter vector containing the promoter region of HK-2 together with SALL4 siRNA or SALL4 overexpressing plasmid as indicated. At 48 h after transfection, the cells were collected and lysed. The luciferase activity was detected by using the dual luciferase assay kit (Promega, Madison, WI, USA).

### Microarray analysis

Total RNAs were isolated from control and SALL4-targeting shRNA transfected MGC-803 cells (3 samples/group). TRIzol reagent (Invitrogen). RNA samples were then used to generate Cyanine-3-CTP(Cy3)-labeled cRNA targets for the gene expression analysis by using Agilent Human lncRNA microarray v2.0 4 × 180 K (OE Biotech, Shanghai, China). The labeled cRNA targets were then hybridized in the slides. After hybridization, slides were scanned on the Agilent Scanner G2505C (Agilent Technologies). Data were extracted with Feature Extraction software 10.7.1.1 (Agilent technologies). Genespring software 12.5 (Agilent technologies) were employed to finish the basic analysis with the raw data. Significant differential expressed transcripts were screened by fold change ≥ 2 or ≤ −2 and *p* value ≤ 0.05. Afterwards, Gene Ontology (GO) and Kyoto Encyclopedia of Genes and Genomes (KEGG) analysis were applied to determine the roles of these differentially expressed mRNAs.

### Chromatin immunoprecipitation assay

The chromatin immunoprecipitation (ChIP) assay was performed in MGC-803 and SGC-7901 cells by using a commercial kit (Millipore, Darmstadt, Germany). After cross-linking with 1% formaldehyde at 37 °C for 10 min, the cells were harvested in sodium dodecyl sulfate lysis buffer and the DNA was shredded to fragments of 200 bp by sonication. The pre-cleared chromatin was incubated with 1 µg anti-SALL4 (ab29112, Abcam) or non-specific IgG overnight. Protein G-agarose beads were added and incubated at 4 °C for 1 h. After reversing the cross-links, the DNA was isolated and used for PCR. The information of the sequences of ChIP primers are listed in Additional file 2.

### Transwell migration assay

The transfected cells were plated into the upper chamber (8 µm) at a density of 1 × 10^5^ cells/well in serum-free medium. The lower chamber was filled with 600 µL complete medium. After incubation at 37 °C in 5% CO_2_ for 12 h, the cells remaining at the upper surface of the membrane were removed with a cotton swab. The cells that migrated through the 8 µm sized pores and adhered to the lower surface of the membranes were fixed with 4% paraformaldehyde, stained with crystal violet and photographed under a light microscope.

### Matrigel invasion assay

The matrigel (BD Biosciences, San Jose, CA, USA) was diluted with serum-free medium (1:3) and 50 µL of the diluted matrigel were added into the upper chamber followed by incubation at 37 °C for 1 h. The transfected cells suspended in serum-free medium were seeded into the upper chamber at a density of 2 × 10^5^ cells/well. The lower chamber was filled with 600 µL complete medium. The cells were allowed to invade into the lower membrane through matrigel at 37 °C for 24 h. Subsequently, the invaded cells were fixed with 4% paraformaldehyde, stained with crystal violet and photographed under a light microscope.

### Cell counting and colony formation assays

The transfected cells were seeded into 24-well plate (1 × 10^4^ cells/well) and cultured under standard conditions. Cells were collected and counted at the indicated time points. The transfected cells were seeded into 6-well plates at a density of 1000 cells per well. After continuous incubation for 10 days, the cells were fixed with 4% paraformaldehyde and stained with 1% crystal violet for 15 min. All the experiments were performed in triplicates.

### RNA extraction and quantitative real-time polymerase chain reaction

Total RNA was extracted from the cells by using Trizol reagent (Life Technologies) and one microgram of RNA was reverse transcribed to cDNA by using reverse transcriptase (Vazyme, Nanjing, China). Quantitative real-time polymerase chain reaction (qRT-PCR) was performed by using a SYBR Green I real-time detection kit (Cwbio, Beijing, China) on a Bio-Rad CFX96 detection system. The relative gene expression was normalized to β-actin. The primers specific for target genes were listed in Additional file 2.

### Glucose uptake, lactate production, lactate dehydrogenase activity, ATP level and hexokinase activity

To detect glucose uptake, lactate production, lactate dehydrogenase activity, ATP level, and hexokinase activity in gastric cancer cells, the Glucose Assay Kit (Applygen, Beijing, China), Lactate Acid Assay Kit (Jiancheng Bioengineering Institute, Nanjing, China), Lactate Dehydrogenase Activity Assay Kit (Jiancheng bioengineering institute), luciferase-based ATP Assay Kit (Beyotime, Shanghai, China), and Hexokinase Activity Assay Kit (Comin, Suzhou, China) were used according to the manufacturer’s protocols. All values were normalized to cell number or total protein levels.

### Western blot analysis

The cells were washed twice with PBS and lysed with RIPA buffer containing 1% protease inhibitors. Equal amounts of proteins were separated on 12% sodium dodecyl sulfate–polyacrylamide gels and transferred onto polyvinylidene fluoride membranes, followed by blocking with 5% nonfat milk for 1 h. The membranes were incubated with primary antibodies overnight at 4 °C. The following primary antibodies were used: anti-SALL4 (1:500, ab29112, Abcam), anti-HK-2 (1:1000, 2867T, Cell Signaling Technology), anti-LDHA (1:1000, 3582T, Cell Signaling Technology), anti-PKM2 (1:1000, 4053T, Cell Signaling Technology), anti-β-actin (1:1000, 4970T, Cell Signaling Technology). After incubation with the secondary antibodies (Bioworld Technology) at 37 °C for 1 h, the bands were visualized with a chemiluminescent detection system.

### Animal study

Male BALB/c nude mice aged 4 weeks were purchased from the Model Animal Research Cancer of Nanjing University (Nanjing, China) and maintained in accordance with the institutional policies. The mice were randomly grouped (five mice/group) as indicated. Cells (2 × 10^6^ per mice) suspended in 100 µL of phosphate-buffered saline were implanted subcutaneously or intraperitoneally into the mice. At 6 weeks after injection, the mice were sacrificed. The protocol was approved by the Animal Use and Care Committee of Jiangsu University.

### Statistical analysis

All the results were expressed as mean ± SD. Statistical analyses were performed using Student’s t-test with GraphPad Prism Version 7.0 software (Graphpad Software, La Jolla, CA, USA). *P *< 0.05 was considered as statistically significant.

## Results

### SALL4 gene silencing suppresses while gene overexpression promotes glycolysis in gastric cancer cells

**Fig. 1:**
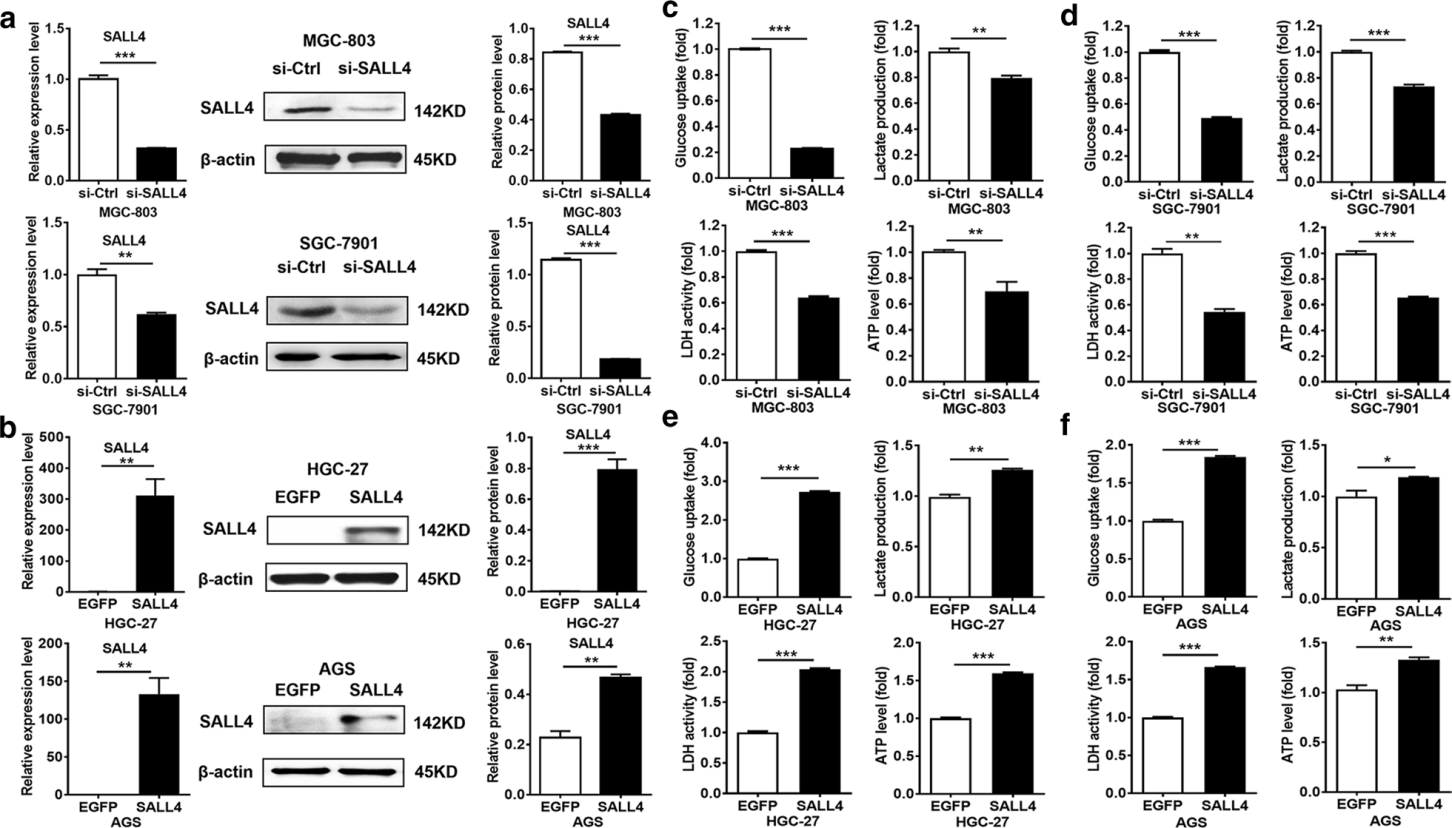
SALL4 gene silencing suppresses while gene overexpression promotes the glycolytic phenotype in gastric cancer cells. **a, b** The efficiency of SALL4 knockdown and overexpression was verified by qRT-PCR and western blot. **c, d** The levels of glucose uptake, lactate production, lactate dehydrogenase activity, and ATP level in control and SALL4 knockdown MGC-803 and SGC-7901 cells. **e, f** The changes in levels of glucose uptake, lactate production, lactate dehydrogenase activity and ATP level in control and SALL4 overexpressing HGC-27 and AGS cells. Vertical bars represented SD of the mean values (n = 3); *P < 0.05, **P < 0.01, ***P < 0.001

We first wanted to know whether SALL4 modulates the glycolytic phenotype of cultured gastric cancer cells. Since the expression of SALL4 in AGS cells is relatively lower than MGC-803 and SGC-7901 cells but is higher than HGC-27 cells, we chose to use MGC-803 and SGC-7901 cells for knockdown study and HGC-27 and AGS cells for overexpression study. We knocked down SALL4 in MGC-803 and SGC-7901 cells by siRNA and overexpressed SALL4 in HGC-27 and AGS cells by plasmid transfection. The efficiency of gene silencing and overexpression was verified by qRT-PCR and western blot (Fig. 1a, b). Then, we compared glucose uptake, lactate production, lactate dehydrogenase activity and ATP level in gastric cancer cells with SALL4 knockdown or overexpression. As shown in Fig. 1c, d, SALL4 knockdown decreased glucose uptake, lactate production, lactate dehydrogenase activity and ATP level in MGC-803 and SGC-7901 cells. On the contrary, SALL4 overexpression increased glucose uptake, lactate production, lactate dehydrogenase activity and ATP level in HGC-27 and AGS cells (Fig. 1e, f). Taken together, these findings indicate that SALL4 regulates glycolysis in gastric cancer cells.

### SALL4 regulates the expression of glycolytic genes in gastric cancer cells

**Fig. 2:**
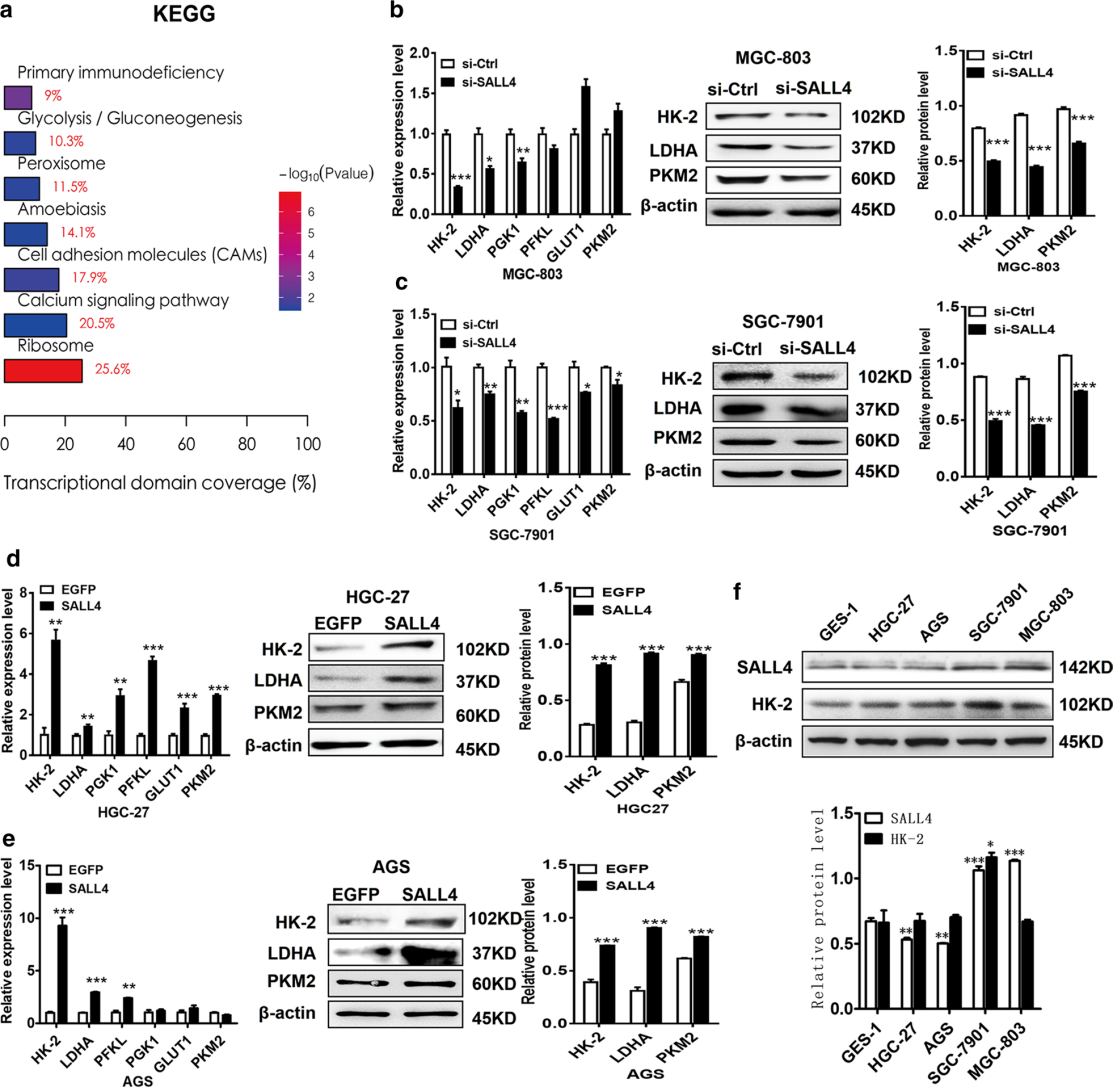
SALL4 regulates the expression of glycolytic genes in gastric cancer cells. **a** The affected signaling pathways between sh-Ctrl- and sh-SALL4-transfected MGC-803 cells were determined by KEGG analysis. **b, c** The expression of glycolytic related genes and proteins in control and SALL4 knockdown MGC-803 and SGC-7901 cells was detected by qRT-PCR and western blot. **d, e** The expression of glycolytic related genes and proteins in control and SALL4 overexpressing HGC-27 and AGS cells was detected by qRT-PCR and western blot. **f.** The protein levels of SALL4 and HK-2 in GES-1, HGC-27, AGS, SGC-7901 and MGC-803 cells were detected by western blot. Vertical bars represented SD of the mean values (n = 3); *P < 0.05, **P < 0.01, ***P < 0.001

To identify the downstream genes and signaling pathway regulated by SALL4, we have previously performed a microarray to compare the differentially expressed genes between control and SALL4 knockdown gastric cancer cells [23]. The results of KEGG analyses showed that the differentially expressed genes were associated with cell adhesion (*P *= 0.02), glycolysis/gluconeogenesis (*P *= 0.03), and calcium signaling pathway (*P *= 0.04) (Fig. 2a). The regulation of cell adhesion molecules such as integrin by SALL4 has been previously reported in breast cancer cells [24]. The differentially expressed genes involved in glycolysis/gluconeogenesis include hexokinase, phosphofructokinase, lactate dehydrogenase and enolase, among others. Inspired by the microarray data and the previous studies [6, 25], we then detected the expression of several key glycolytic genes in control, SALL4 knockdown, and SALL4-overexpressing gastric cancer cells, including HK-2, lactate dehydrogenase A (LDHA), phosphofructokinase (PFKL), phosphoglycerate kinase 1 (PGK1), Glucose transporter type 1 (GLUT1), and pyruvate kinase M2 (PKM2). As shown in Fig. 2b, c, SALL4 knockdown reduced the expression levels of HK-2, LDHA and PGK1 genes in MGC-803 and SGC-7901 cells (Fig. 2c). SALL4 knockdown also decreased the expression of HK-2 and LDHA proteins in MGC-803 and SGC-7901 cells (Fig. 2b, c). On the contrary, SALL4 overexpression by plasmid transfection increased the expression of HK-2, LDHA, PFKL genes in HGC-27 cells and AGS cells (Fig. 2d, e). SALL4 overexpression also increased the expression of HK-2 and LDHA proteins in HGC-27 and AGS cells (Fig. 2d, e). The gastric cancer cells with high levels of SALL4 seem to also have increased expression of HK-2 (Fig. 2f). Intriguingly, HK-2 has been previously shown to be downregulated in the microarray data of another study whereby SALL4 is knocked down in hepatocellular carcinoma (HCC) cells [26]. Considering that HK-2 is the first rate-limiting enzyme of glycolysis and it showed the most significant change after SALL4 knockdown and overexpression in gastric cancer cells, we hypothesized that SALL4 might promote glycolysis through the regulation of HK-2 in gastric cancer cells.

### HK-2 is identified as a downstream target of SALL4

**Fig. 3:**
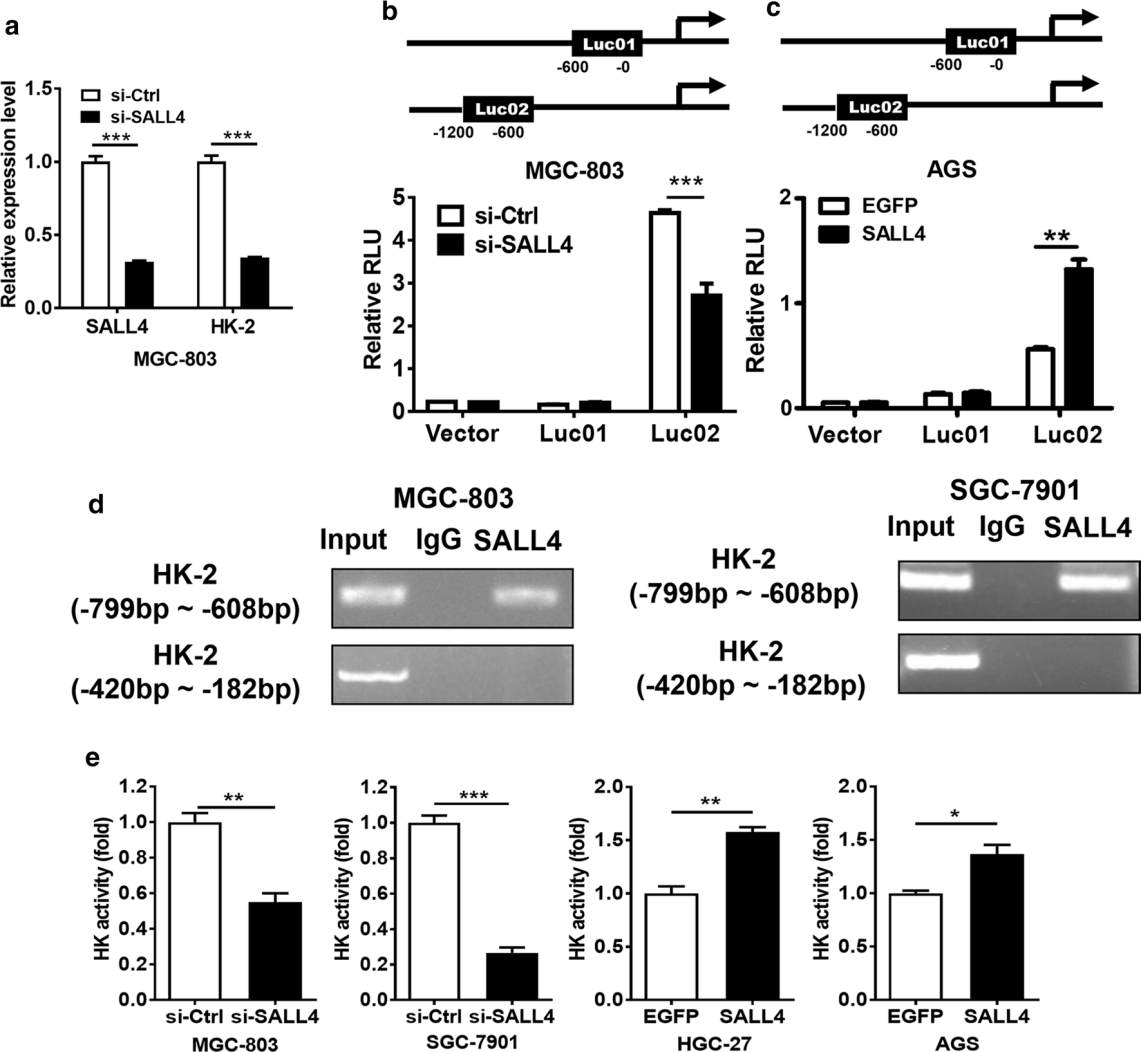
HK-2 is identified as a downstream target of SALL4. **a** Verification of microarray results for differentially expressed genes between control and SALL4 knockdown MGC-803 cells. **b, c** Luciferase reporter assays for the relative luciferase activity of HK-2 gene promoter. **d** ChIP assay for the binding of SALL4 to the promoter region of HK-2 gene. **e** The changes in levels of hexokinase activity in control and SALL4 knockdown and overexpressing gastric cancer cells. Vertical bars represented SD of the mean values (n = 3); *P < 0.05, **P < 0.01, ***P < 0.001

In consistent with the microarray data, our qRT-PCR results confirmed that HK-2 expression was decreased in SALL4 knockdown MGC-803 cells (Fig. 3a). To test whether SALL4 transcriptionally regulates HK-2 gene expression, we searched up to approximately 1200 bp of the promoter region of HK-2 gene for putative SALL4 binding sites and constructed two promoter luciferase reporters. The results of luciferase reporter assay showed that SALL4 knockdown downregulated while SALL4 overexpression upregulated the luciferase activity of HK-2 gene promoter (Fig. 3b, c). The -1200 to -600 bp region in HK-2 gene promoter was critical for SALL4-mediated transactivation. We then performed ChIP assays to test the binding of SALL4 protein to HK-2 gene promoter in MGC-803 and SGC-7901 cells. ChIP assay results showed that SALL4 could bind to the -799 to -608 bp region of the promoter of HK-2 gene (Fig. 3d). There was no binding between SALL4 and the -420 to -182 bp region of the promoter of HK-2 gene (Fig. 3d). Consistent with these results, we observed a decrease of hexokinase activity in SALL4 knockdown MGC-803 and SGC-7901 cells and an increase of hexokinase activity in SALL4- overexpressing HGC-27 and AGS cells (Fig. 3e). Taken together, these results suggest that SALL4 regulates the expression and activity of HK-2 in gastric cancer cells.

### HK-2 inhibition abrogates the induction of glycolysis in gastric cancer cells by SALL4 overexpression

**Fig. 4:**
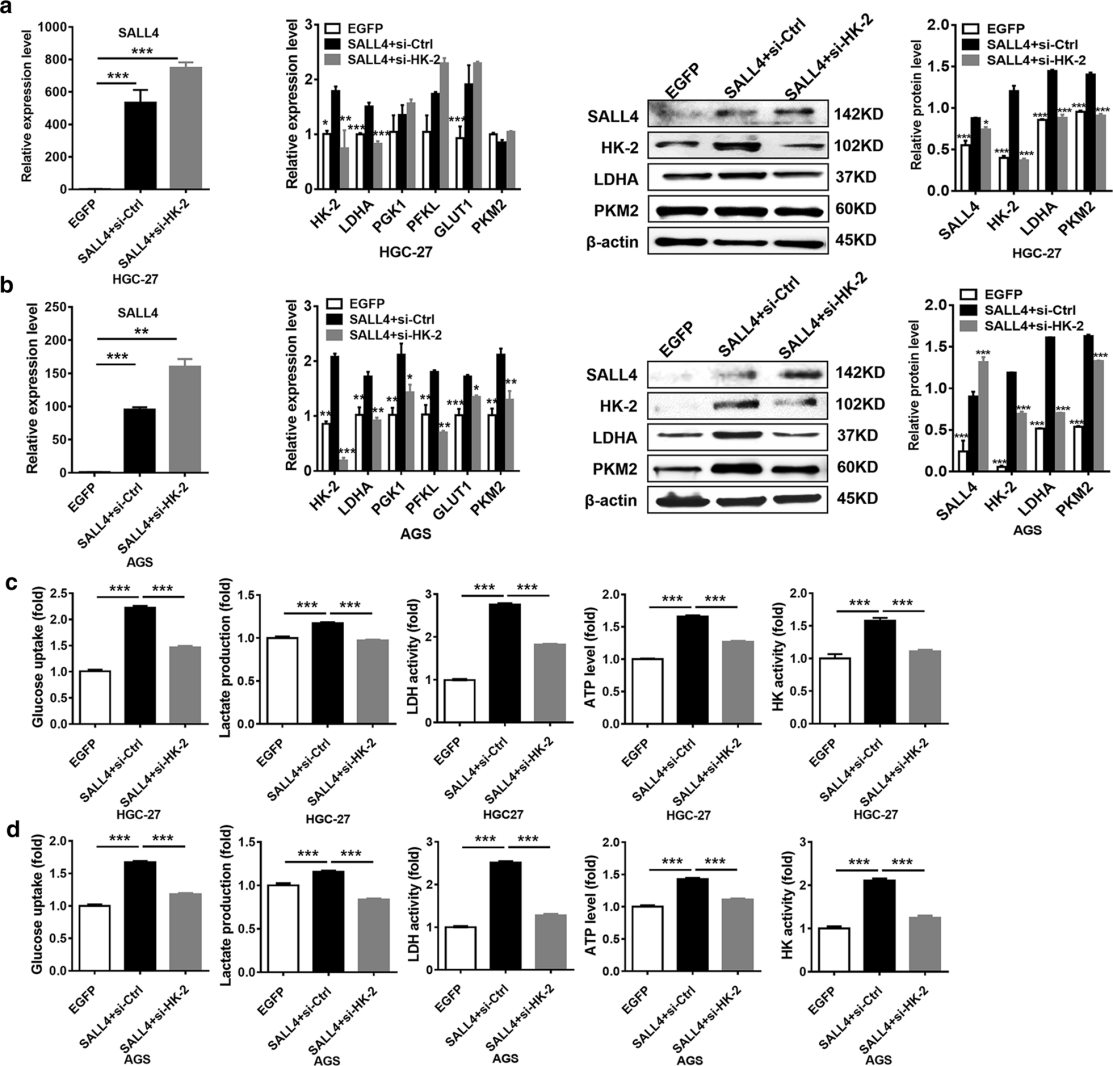
HK-2 mediates the promotion of glycolysis by SALL4 in gastric cancer cells. **a, b** qRT-PCR and western blot analyzed the expression of SALL4, HK-2 and glycolytic related genes and proteins in SALL4-overexpressing HGC-27 cells and AGS cells with or without HK-2 knockdown. **c, d** The levels of glucose uptake, lactate production, lactate dehydrogenase activity, ATP level, and hexokinase activity in SALL4-overexpressing HGC-27 cells and AGS cells with or without HK-2 knockdown. Vertical bars represented SD of the mean values (n = 3); *P < 0.05, **P < 0.01, ***P < 0.001

To further confirm that HK-2 is a direct target of SALL4, we determined the effect of SALL4 on glycolysis in HK-2 knockdown HGC-27 and AGS cells. We co-transfected HGC-27 and AGS cells with SALL4-overexpressing plasmid and HK-2 siRNA. As shown in Fig. 4a, b, the upregulation of HK-2 by SALL4 was reversed by HK-2 interference. Consistent with these results, SALL4 overexpression could not further increase the levels of glycolysis, including glucose uptake, lactate production, lactate dehydrogenase activity, ATP level and hexokinase activity in HGC-27 and AGS cells when HK-2 expression was interfered (Fig. 4c, d). These results indicate that HK-2 inhibition abrogates the promotion of glycolysis by SALL4 in gastric cancer cells.

### HK-2 inhibition disturbs the promotion of gastric cancer cell proliferation, migration and invasion by SALL4 overexpression

**Fig. 5:**
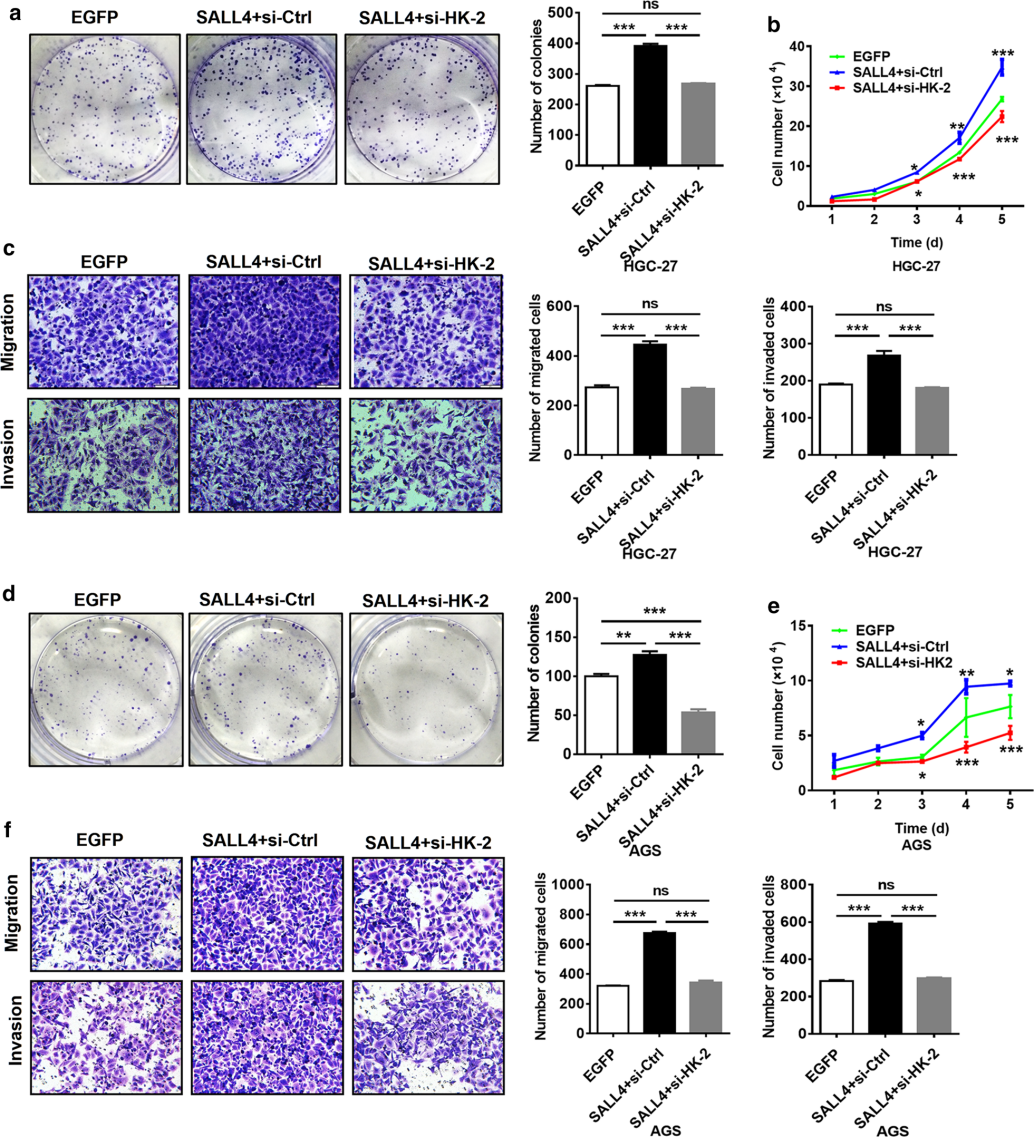
HK-2 mediates the promoting role of SALL4 in gastric cancer cells proliferation, migration and invasion. **a, d** Cell colony formation assay for the growth ability of SALL4-overexpressing HGC-27 and AGS cells with or without HK-2 knockdown. **b, e** Cell counting assay for the growth ability of SALL4-overexpressing HGC-27 and AGS cells with or without HK-2 knockdown. **c, f** Transwell migration and matrigel invasion assays for the migration and invasion abilities of SALL4-overexpressing HGC-27 and AGS cells with or without HK-2 knockdown. Vertical bars represented SD of the mean values (n = 3); *P < 0.05, **P < 0.01, ***P < 0.001

Glycolysis is closely related to many malignant behaviors of tumor cells. The previous studies have shown that SALL4 overexpression promotes gastric cancer cell proliferation, migration, and invasion [21–23]. Therefore, we wanted to know whether HK-2 is related to these effects of SALL4. We overexpressed SALL4 in HGC-27 and AGS cells in the presence or absence of HK-2 interference. We found that the proliferation, migration and invasion abilities of gastric cancer cells, which had been enhanced by SALL4 overexpression, were weakened after interfering with HK-2 expression (Fig. 5). Therefore, SALL4 may maintain the malignant phenotypes of gastric cancer cells by regulating HK-2-mediated glycolysis.

### SALL4 knockdown inhibits gastric cancer growth and metastasis though the downregulation HK-2

**Fig. 6:**
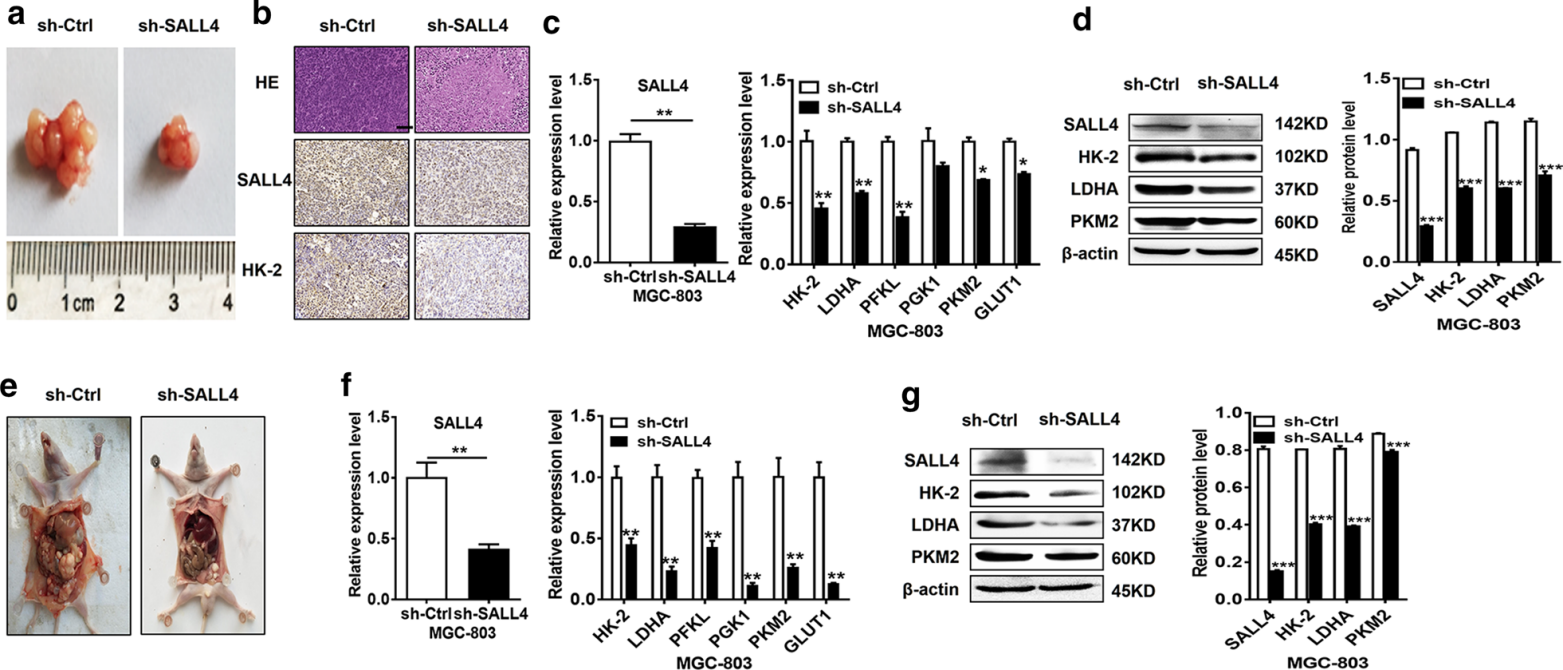
SALL4 knockdown inhibits glycolysis and suppresses gastric cancer growth and metastasis though the downregulation HK-2 in vivo. **a, e** Representative images of tumors from mice injected with control and SALL4 knockdown MGC-803 cells subcutaneously and intraperitoneally (n = 5 per group). **b** Representative images of HE staining and immunohistochemical staining of SALL4 and HK-2. **c, d, f, g** qRT-PCR and western blot analyses of glycolysis-related genes and proteins in tumors from mice injected with control and SALL4 knockdown MGC-803 cells. Vertical bars represented SD of the mean values (n = 3); *P < 0.05, **P < 0.01, ***P < 0.001. Scale bar: 50 μm

To verify whether the regulation of HK-2 by SALL4 is critical for gastric carcinogenesis, we established subcutaneous tumor-bearing and peritoneal metastasis tumor mouse models by using control and SALL4 knockdown gastric cancer cells. Consistent with our previous reports, SALL4 knockdown inhibited tumor growth in both models (Fig. 6a, e). Tumor tissues from control and SALL4 knockdown groups were collected for the detection of gene and protein expression. The results of qRT-PCR, western blot, and immunohistochemistry showed that HK-2 expression was decreased in SALL4 knockdown group compared to control group in both models (Fig. 6b, c, d, f and g), suggesting that SALL4 knockdown decreases HK-2 expression, which in turn suppresses glycolysis in gastric cancer cells and disturbs cancer progression.

## Discussion

Aerobic glycolysis is the main form of energy metabolism in tumor cells [27, 28] and is closely related to cancer development and progression [29, 30]. In recent years, increasing studies suggest that the aberrant activation of multiple signaling pathways [31–33] and transcription factors could promote Warburg effect through the regulation of key glycolytic genes such as GLUT1 and LDHA [3–7]. Increased glycolysis leads to enhanced tumor growth, angiogenesis, metastasis, and drug resistance in various cancers [31, 34, 35]. Moreover, many studies have shown that down-regulation of glycolytic genes or inhibition of glycolysis could suppress tumor growth [36–38]. Increased levels or activities of three rate-limiting enzymes involved in glycolysis, including HK-2, accelerates gastric cancer progression and leads to poor prognosis in cancer patients [39]. In mammals, hexokinase has four subtypes, which are encoded by different genes. HK-2 overexpression is positively related to the high level of tumor glycolysis and the low overall survival of tumor patients [40–42]. In addition to glycolysis, HK-2 is also involved in cell proliferation, apoptosis, and autophagy. For instance, HK-2 could bind to and interact with mitochondrial voltage-dependent anion channels, stabilize the mitochondrial membrane, prevent pro-apoptotic factors from binding to it, and thus inhibit apoptosis [43], suggesting a critical role of HK-2 in caner development and progression through both glycolysis-dependent and -independent mechanisms.

**Fig. 7:**
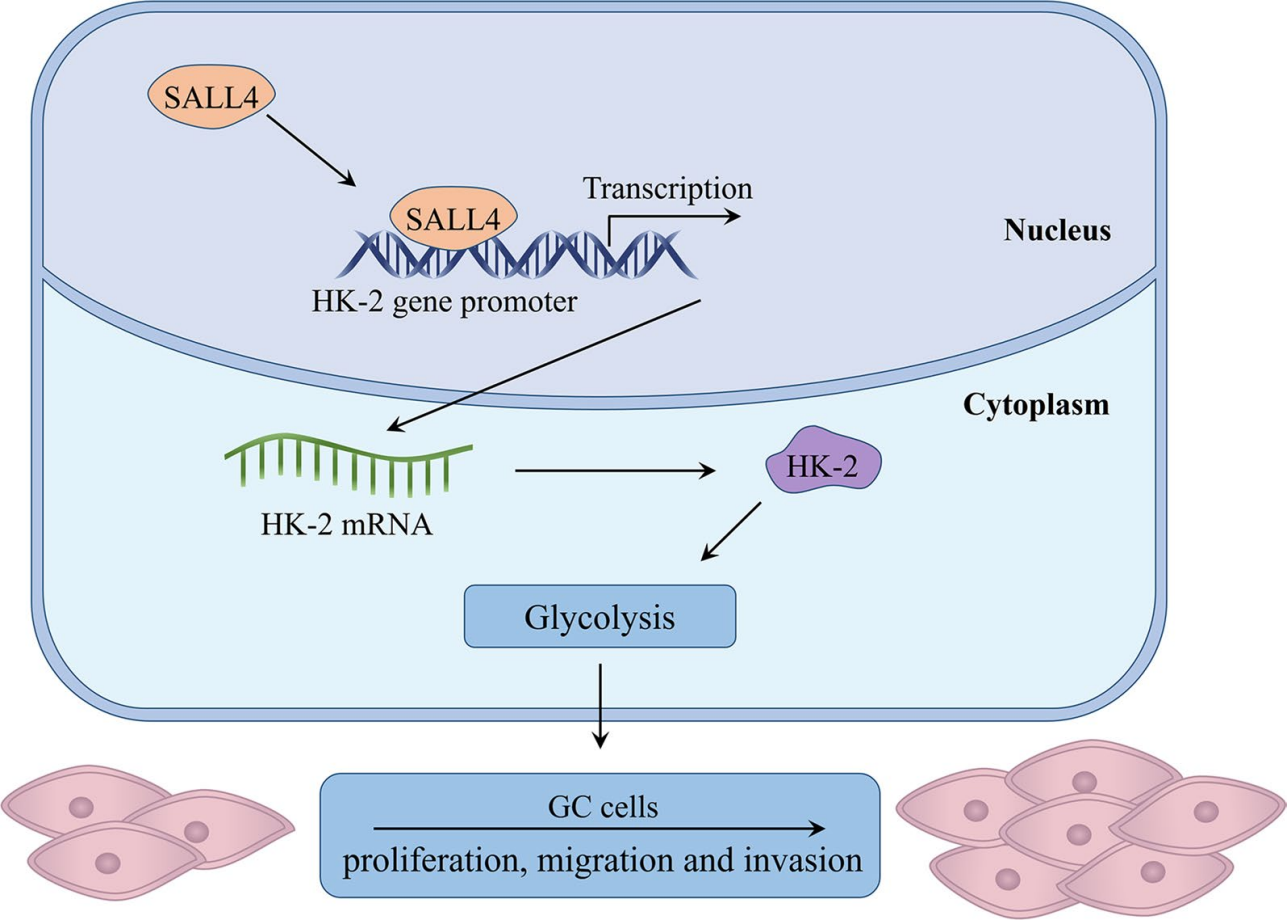
The proposed model for SALL4 regulation of glycolysis in gastric cancer. SALL4 upregulates HK-2 transcription and induces glycolysis in a HK-2 dependent manner, thus promoting the proliferation, migration and invasion of gastric cancer cells

HK-2 expression is regulated at both transcriptional and post-transcriptional levels [44–46]. Several studies have shown that c-Myc binds to the regulatory region of HK-2 gene and plays a pivotal role in glucose metabolism. The transcription factor BACH1 could activate HK-2 transcription and increase glucose uptake, glycolytic rate, and lactate secretion, thereby stimulating glycolysis-dependent metastasis of human lung cancer cells. In epithelial ovarian cancer, FOXM1 promotes reprogramming of glucose metabolism in cancer cells via activation of HK-2 and GLUT1 transcription [25]. The regulation of HK-2 by miRNAs has also been widely reported [36, 41]. In this study, we found that SALL4 could also regulate the transcription of HK-2, thereby promoting glycolysis and gastric cancer progression (Fig. 7). The transcription factor SALL4 has been extensively studied in human cancers. Increasing evidence suggest that SALL4 promotes tumor growth, metastasis, and therapy resistance through the regulation of c-Myc [13], TGF-β1 [23], ATP-binding cassette (ABC) [15], among others. Li et al. demonstrate that SIX1 interacts with histone acetyltransferase HBO1 and AIB1 to induce the expression of glycolytic genes and enhance glycolysis, which ultimately promotes cell malignant transformation and cancer development [6]. Since the consensus binding sequence for SALL4 has not been identified, we analyzed the promoter region of HK-2 gene and found that SALL4 could bind to the -799 to -608 bp region of the promoter of HK-2 gene. Thus, SALL4 may bind to this region and recruit other factors (such as HDACs) to enter into this site, opening the chromatin structure of HK-2 gene and initiating transcription. In addition, this region harbors the binding sites for other transcription factors such as Oct4 and Sox2. Whether other factors cooperate with SALL4 to regulate HK-2 transcription warrants further investigation.

Accumulating studies suggest that oncogenes promote gastric cancer progression through glycolysis. For example, AhpC/TSA antioxidant enzyme domain containing 1 (AAED1) enhances the proliferation of gastric cancer cells by promoting glycolysis [47]. Kim et al. demonstrate that SALL4 could induce drug resistance by promoting glycolysis [18]. We found that SALL4 promoted glycolysis by enhancing the expression of HK-2 and interfere with HK-2 expression inhibited the promoting role of SALL4 in gastric cancer cell proliferation, migration and invasion. Shi et al. demonstrate that B7-H3 promotes aerobic glycolysis by promoting HK-2 expression and HK-2 is a key mediator of B7-H3-induced chemoresistance [37]. In consistence with these findings, our results showed that SALL4, at least in part, promoted gastric cancer progression by regulating HK-2-mediated glycolysis. However, it could not be excluded that SALL4 may regulate glycolysis and participate in gastric cancer progression through other mechanisms.

## Conclusion

In conclusion, our findings show that SALL4 induces glycolysis via the upregulation of HK-2, thus promoting the proliferation, migration and invasion of gastric cancer cells (Fig. 7). Our study not only reveals a new mechanism for the oncogenic roles of SALL4 in cancer, but also provides evidence for the potential of SALL4 as a therapeutic target for gastric cancer.

## Supplementary information

**Additional file 1:Table S1.** Sequences of shRNA and siRNA

**Additional file 2: Table S2.** Sequences of PCR primers for target gene detection

## Data Availability

All data generated or analyzed during this study are included in this article.

## Abbreviations

AAED1: AhpC/TSA antioxidant enzyme domain containing 1
ABC: ATP-binding cassette
ChIP: Chromatin immunoprecipitation
Cy3: Cyanine-3-CTP
FBS: Fetal bovine serum
GLUT1: Glucose transporter 1
GO: Gene ontology
HCC: Hepatocellular carcinoma
HK-2: Hexokinase II
HIF-1α: Hypoxia inducible factor-1α
HP1α: Heterochromatin protein 1α
KEGG: Kyoto Encyclopedia of Genes and Genomes
LDHA: Lactate dehydrogenase A
MOI: Multiplicity of infection
PFKL: 6-phosphofructokinase, liver type
PGK1: Phosphoglycerate kinase 1
PKM2: Pyruvate kinase M2
qRT-PCR: Quantitative real-time polymerase chain reaction
SIX1: Sine oculis homeobox 1
