# Supplementary material for: SALL4 promotes gastric cancer progression via hexokinase II mediated glycolysis
Source: Cancer Cell Int. 2020 May 24;20:188. doi: 10.1186/s12935-020-01275-y (PMC7247129; doi:10.1186/s12935-020-01275-y)
Supplement: Supplementary file 1 — Additional file 1:Table S1. Sequences of shRNA and siRNA [file 12935_2020_1275_MOESM1_ESM.docx]

| **Additional file 1: Sequences of shRNA and siRNA** | |
| --- | --- |
| **shRNA** | **Target sequence** |
| sh-Ctrl | 5'-TTCTCCGAACGTGTCACGT-3' |
| sh-SALL4 | 5'-GACCTATGTCAAGGTTGAA-3' |
| si-Ctrl | F:5'-UUCUCCGAACGUGUCACGUTT-3' |
|  | R:5'-ACGUGACACGUUCGGAGAATT-3' |
| si-SALL4 | F:5'-GUCUCUGGAUGCCUUGAAATT-3' |
|  | R:5'-UUUCAAGGCAUCCAGAGACTT-3' |
| si-HK-2 | F:5'-GAGAAUCAGAUCUAUGCCATT-3' |
|  | R:5'-UGGCAUAGAUCUGAUUCUCTT-3' |
