# Supplementary material for: SALL4 promotes gastric cancer progression via hexokinase II mediated glycolysis
Source: Cancer Cell Int. 2020 May 24;20:188. doi: 10.1186/s12935-020-01275-y (PMC7247129; doi:10.1186/s12935-020-01275-y)
Supplement: Supplementary file 2 — Additional file 2: Table S2. Sequences of PCR primers for target gene detection [file 12935_2020_1275_MOESM2_ESM.docx]

| **Additional file 2: Sequences of PCR primers for target gene detection** | |
| --- | --- |
| **Gene** | **Primer sequence** |
| SALL4 | F:5'-TCGATGGCCAACTTCCTTC-3' |
|  | R:5'-GAGCGGACTCACACTGGAGA-3' |
| β-actin | F:5'-CACGAAACTACCTTCAACTCC-3' |
|  | R:5'-CATACTCCTGCTTGCTGATC-3' |
| HK-2 | F:5'-GCCATCCTGCAACACTTAGGGCTTGAG-3' |
|  | R:5'-GTGAGGATGTAGCTTGTAGAGGGTCCC-3' |
| LDHA | F:5'-ATGGCAACTCTAAAGGATCA-3' |
|  | R:5'-GCAACTTGCAGTTCGGGC-3' |
| PGK1 | F:5'-ATGTCGCTTTCTAACAAGCTGA-3' |
|  | R:5'-GCGGAGGTTCTCCAGCA-3' |
| PFKL | F:5'-GGAGAAGCTGCGCGAGGTTTAC-3' |
|  | R:5'-ATTGTGCCAGCATCTTCAGCATGAG-3' |
| GLUT1 | F:5'-CATCCCATGGTTCATCGTGGCTGAACT-3' |
|  | R:5'-GAAGTAGGTGAAGATGAAGAACAGAAC-3' |
| PKM2 | F:5'-GCCCGTGAGGCAGAGGCTGC-3' |
|  | R:5'-TGGTGAGGACGATTATGGCCC-3' |
| HK-2 | F:5'-TGAGGGTGAGTGGCAAGA-3' |
| (-799bp~-608bp) | R:5'-CTGGAGTTGCAGGCTTATTT-3' |
| HK-2 | F:5'-GGGAGTGAGGTTAGCCAGAA-3' |
| (-420bp~-182bp) | R:5'-CCTCAACCCTCCTTCCCTAT-3' |
